# Characteristics of ammonia, acid gases, and PM_2.5_ for three typical land-use types in the North China Plain

**DOI:** 10.1007/s11356-015-5648-3

**Published:** 2015-10-27

**Authors:** Wen Xu, Qinghua Wu, Xuejun Liu, Aohan Tang, Anthony J. Dore, Mathew R. Heal

**Affiliations:** College of Resources and Environmental Sciences, China Agricultural University, Beijing, 100193 China; Centre for Ecology and Hydrology, Edinburgh, Bush Estate, Penicuik, Midlothian, EH26 0QB UK; School of Chemistry, The University of Edinburgh, David Brewster Road, Edinburgh, EH9 3FJ UK

**Keywords:** Air pollution, Reactive N, PM_2.5_, Control strategies, Chemical characteristics, The North China Plain

## Abstract

Air pollution is one of the most serious environmental problems in China due to its rapid economic development alongside a very large consumption of fossil fuel, particularly in the North China Plain (NCP). During the period 2011–2014, we integrated active and passive sampling methods to perform continuous measurements of NH_3_, HNO_3_, NO_2_, and PM_2.5_ at two urban, one suburban, and two rural sites in the NCP. The annual average concentrations of NH_3_, NO_2_, and HNO_3_ across the five sites were in the ranges 8.5–23.0, 22.2–50.5, and 5.5–9.7 μg m^−3^, respectively, showing no significant spatial differences for NH_3_ and HNO_3_ but significantly higher NO_2_ concentration at the urban sites. At each site, annual average concentrations of NH_3_ and NO_2_ showed increasing and decreasing trends, respectively, while there was no obvious trend in annual HNO_3_ concentrations. Daily PM_2.5_ concentrations ranged from 11.8 to 621.0 μg m^−3^ at the urban site, from 19.8 to 692.9 μg m^−3^ at the suburban site, and from 23.9 to 754.5 μg m^−3^ at the two rural sites, with more than 70 % of sampling days exceeding 75 μg m^−3^. Concentrations of water-soluble ions in PM_2.5_ ranked differently between the non-rural and rural sites. The three dominant ions were NH_4_^+^, NO_3_^−^, and SO_4_^2−^ and mainly existed as (NH_4_)_2_SO_4_, NH_4_HSO_4_, and NH_4_NO_3_, and their concentrations averaged 48.6 ± 44.9, 41.2 ± 40.8, and 49.6 ± 35.9 μg m^−3^ at the urban, suburban, and rural sites, respectively. Ion balance calculations indicated that PM_2.5_ was neutral at the non-rural sites but acidic at the rural sites. Seasonal variations of the gases and aerosols exhibited different patterns, depending on source emission strength and meteorological conditions. Our results suggest that a feasible pathway to control PM_2.5_ pollution in the NCP should target ammonia and acid gases together.

**Electronic supplementary material:**

The online version of this article (doi:10.1007/s11356-015-5648-3) contains supplementary material, which is available to authorized users.

## Introduction

In China, the atmospheric environment has been greatly affected over recent decades by various anthropogenic factors, such as a dramatic economic rise, rapid industrial development, population growth, and construction and demolition projects. The increase of traffic flow is also of central importance. As a consequence, complex air pollution events characterized by regional photochemical smog and haze occur frequently in many regions of China (Wang et al. 2014a), arousing increasing attention from the private citizen as well as environmental scientists and policy makers. The smog and haze largely result from high levels of particulate matter (PM), especially PM_2.5_ (particular matter less than 2.5 μm), which limits atmospheric visibility by light extinction (absorption and scattering) (Sun et al. 2006; Wang et al. 2012a). Several studies focusing on health effects have revealed associations between PM pollution and morbidity and mortality, including in the USA (Doninici et al. 2014) and China (Guo et al. 2009; Wu et al. 2010). It has been estimated that 350,000–400,000 premature deaths can be ascribed to ambient air pollution in China, and the economic burden of premature mortality and morbidity was conservatively estimated at approximately 157 billion RMB (1.16 % of the GDP) in 2003 (Zhang and Smith 2007; WB 2007).

Airborne PM_2.5_ can be directly emitted by anthropogenic sources or generated by gas-to-particle conversion (secondary PM) (Watson 2002). The primary precursors for formation of ammonium sulfate (or bisulfate) and ammonium nitrate are NH_3_, SO_2_, and NO_*x*_ (NO + NO_2_). Atmospheric NH_3_ is emitted primarily from livestock wastes and volatilization of N fertilizers. Other sources include biomass burning, excreta of human and pets, and wastewater (Clarisse et al. 2009). NO_*x*_ and SO_2_ are mainly derived from combustion processes and are subsequently oxidized to HNO_3_ and H_2_SO_4_ in the atmosphere (Sharma et al. 2007).

In order to prevent further deterioration of air quality, China has made tremendous efforts since 2005. For example, the 11th Five-Year Plan (FYP) (2006–2010) for national environmental protection required the reduction of annual SO_2_ emissions in 2010 by 10 % from its 2005 level, which required installation of flue-gas desulfurization systems to coal-fired power plants as a primary control measure and a stronger vehicle emissions standard. As a consequence, national SO_2_ emissions decreased by 14.3 % from 2005 to 2010 (MEPC 2011). In the 12th FYP (2011–2015), China is mainly focused on the reduction of national NO_*x*_ emissions by 10 % in 2015 from the 2010 level, as well as controls on SO_2_ and primary particle emissions. To achieve this binding target, China’s Ministry of Environmental Protection (MEP) released a new “emission standard of air pollutants for thermal power plants” (GB 13223-2011) in 2011 to further strengthen the NO_*x*_ controls. Furthermore, a stricter vehicle emissions standard (equivalent to the European Union’s Euro IV standard) was also released in late 2012. Unfortunately, legislation to simultaneously reduce NH_3_ emissions has not been implemented in China. Such legislation is urgently needed given that the estimated health costs associated with NH_3_ emissions were greater than those associated with NO_*x*_ emissions in more than 77 % of provinces in China, particularly in the North China Plain (NCP) (Gu et al. 2014).

The NCP is an intensively managed agricultural and economically developed region, which comprises only 8 % of the total area of China but contributes 40 % of the total national GDP (CSY 2014). The consumption of N fertilizer and energy in the NCP accounted for 35 and 34 % of their respective total national consumption (CSY 2014). This makes the NCP one of the greatest emitters of air pollutants (e.g., NH_3_, NO_*x*_, and SO_2_) nationally and globally (Clarisse et al. 2009; Zhang et al. 2009; Gu et al. 2012; Huang et al. 2012) and a serious PM_2.5_ pollution region in China (Wang et al. 2014b). Some studies have focused on the measurements of atmospheric NO_2_ and NH_3_ at various sites in the NCP (Shen et al. 2009, 2011; Meng et al. 2011; Pan et al. 2012; Luo et al. 2013) and on estimating of emissions of NO_2_ and NH_3_ from anthropogenic sources (Zhang et al. 2010, 2011b). Very few studies in the NCP have considered ambient HNO_3_ measurements (Shen et al. 2009; Luo et al. 2013). PM_2*.*5_ has been systematically analyzed in many studies in the NCP. Most of the studies have provided the general characteristics of the chemical compositions of PM_2.5_ and discussed its seasonal variations, correlations, or sources (Sun et al. 2004; Song et al. 2006). Additionally, the concentration, correlations, sources, or formation of some specific species (e.g., inorganic ions, carbonaceous components, or organic matter) and their spatial variations have been investigated in the NCP (Ianniello et al. 2011; Zhao et al. 2013; Hu et al. 2014). However, few studies have measured NO_*x*_, NH_3_, HNO_3_, and PM_2.5_ simultaneously. In addition, previous work has mainly included short-term studies before the year 2010 and has been limited to single land-use types (e.g., urban areas). In the absence of long-term and simultaneous observations, the characteristics of these air pollutants and their implications cannot be determined accurately.

In the present study, ambient NO_*x*_, NH_3_, and HNO_3_ were continuously monitored at five typical sites (two urban, one suburban, and two rural) in the NCP during the period 2011–2014, and PM_2.5_ was sampled at four of the five sites. The objectives of this study were (1) to characterize spatial, seasonal, and annual variations of concentrations for the measured gases and evaluate their pollution status during the period from 2011 to 2014 and (2) to analyze the concentrations and seasonal variations of PM_2.5_ and its secondary inorganic components over different land-use types. The intention of the study was to provide accurate and current insight into the characteristics of air pollutants and to support interpretation of the effectiveness of major national control policies implemented recently in the NCP.

## Materials and methods

### Sampling sites

**Fig. 1:**
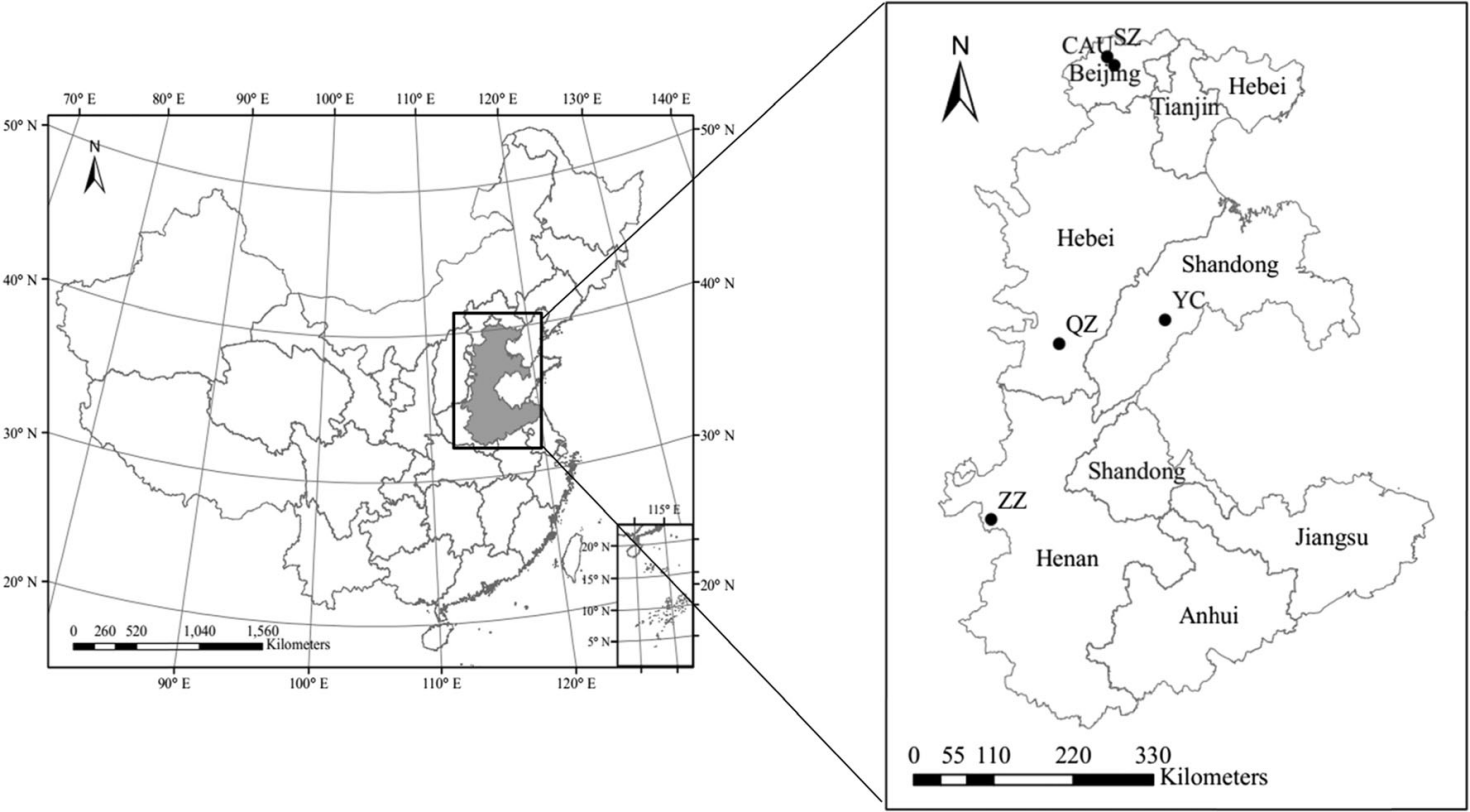
Geographical distribution of the five sampling sites in the North China Plain: urban sites (CAU, ZZ), suburban site (SZ), and rural sites (QZ, YC)

Sampling was conducted between January 2011 and December 2014 at five sites in Beijing and in Henan, Shandong, and Hebei provinces (Fig. 1), which are located in the North China Plain. The area has a typical temperate and monsoonal climate with dry winters and wet summers. The prevailing wind direction is from the southeast in the summer and northwest in the winter.

Two urban sites were at the China Agricultural University (CAU) and Zhengzhou (ZZ), a suburban site was at Shangzhuang (SZ), and two rural sites were at Quzhou (QZ) and Yucheng (YC). The CAU site is located at the west campus of the China Agricultural University (40.02° N, 116.28° E, 55 m above sea level (m.a.s.l.)) which is situated in the northwestern urban area of Beijing. The ZZ site is located at Henan Academy of Agricultural Sciences (34.75 °N, 113.63 °E, 91 m.a.s.l.), which is in the center of Zhengzhou, the capital of Henan province. The SZ site is located at Shuangzhuang Agricultural Experimental Station (40.11° N, 116.20° E, 47 m.a.s.l.) in Shuangzhuang town, which is situated to the northwest of Beijing, about 33 km from the city center. Quzhou (QZ) is a typical rural agriculture dominated site with a recently constructed industrial district, about 60 km northeast of Handan city, Hebei province. The sampling site was located at Quzhou Agricultural Experimental Station (36.78° N, 114.94° E, 37 m.a.s.l.). The YC site is located at Yucheng Experimental Station (36.94° N, 116.63° E, 24 m.a.s.l.), Chinese Academy of Sciences, about 60 km southeast of Dezhou city, Shandong province. The measurement height and period, and other information on sampling sites such as measured species, possible emissions and density of population, are given in Table 1.

**Table 1:** Descriptions of the five monitoring sites and the corresponding measurements in this study

Monitoring site	Species	Monitoring period		Measurement height (m) and underlying surface			Population density (persons km^−2^)^b^	Surrounding environment and possible emission sources
		Gases^a^	PM_2.5_	Gases	PM_2.5_	Surface		
China Agricultural University (CAU)	NH_3_, HNO_3_, NO_2_, PM_2.5_	Jan. 2011–Dec. 2014	Mar. 12–Nov. 2014	2	10	Roof	7616	Densely occupied residences, small-scale urban agriculture, and traffic roads
Shangzhuang (SZ)	NH_3_, HNO_3_, NO_2_, PM_2.5_	Jan. 2011–Dec. 2014	Apr. 12–Nov. 2014	2	2.5	Grass	519	Small towns, traffic roads, and farmland
Quzhou (QZ)	NH_3_, HNO_3_, NO_2_, PM_2.5_	Jan. 2011–Dec. 2014	Apr. 12–Nov. 2014	2	2.5	Grass	606	Small villages, a traffic road, and dense farmland
Yucheng (YC)	NH_3_, HNO_3_, NO_2_, PM_2.5_	Jan. 2013–Dec. 2014	Apr. 13–Nov. 2014	2	2.5	Lawn	495	Small villages, a road, and farmland
Zhengzhou (ZZ)	NH_3_, HNO_3,_ NO_2_	Jan. 2011–Dec. 2014	n.d.	2	–	Grass	17,069	Densely occupied residences and traffic roads

*n.d.* not determined

^a^NH_3_, HNO_3_, and NO_2_

^b^Population density was estimated by dividing population by area of the town/district/county where the monitoring site is located. Population data were sourced from the sixth census of China in 2010 (http://www.stats.gov.cn)

### Sampling methods and chemical analysis

NH_3_ and HNO_3_ samples were collected using the DELTA (DEnuder for Long-Term Atmospheric sampling) system designed by the Centre for Ecology and Hydrology Edinburgh, UK. The DELTA system has been used widely in Europe and described in detail in many previous studies (e.g., Flechard et al. 2011; Luo et al. 2013; Shen et al. 2013). Briefly, the DELTA sampling “train” consists of two potassium carbonate plus glycerol (1 % (*m*/*v*) K_2_CO_3_ + 1 % (*m*/*v*) glycerol in methanol)-coated denuders in series for the simultaneous collection of HNO_3_, followed by two citric acid (5 % (*m*/*v*) citric acid in methanol)-coated denuders for NH_3_. A low-volume pump (D210, TCS Micropumps Ltd., UK) in the DELTA system was used to sample ambient air at a rate of 0.2–0.4 L min^−1^. When the air passes through a denuder filter train, HNO_3_ and NH_3_ in the air are absorbed by the coated chemical solutions in sequence. With a monthly sampling period, the detection limit of the DELTA system for gaseous HNO_3_ and NH_3_ was determined as 0.03 μg HNO_3_ m^−3^ and 0.01 μg NH_3_ m^−3^. Two denuders in series are used for every sample to check capture efficiency for HNO_3_ and NH_3_. When the value is less than 75 %, an imperfectly coated film or some other sampling problems can be assumed to have occurred in the DELTA system (Tang et al. 2009). Across the five sites, collection efficiencies in the first of the two denuders for HNO_3_ and NH_3_ averaged 83.2 % (95 % confidence interval 81.5–84.9 %) and 82.7 % (81.3–84.1 %), respectively, during the entire period. Thus, we can be assured that both of measured gases were effectively captured in both denuders. It should be noted that nitrous acid (HONO) could cause a positive bias in the long-term measurement of HNO_3_ using the DELTA system (Tang et al. 2009). This is of importance in urban areas whereas HONO interference (as well as possible NO_2_ and PAN interference) should be negligible in rural areas. In this study, glycerol was added to the coating of the denuder for HNO_3_ sampling in order to increase adhesion and reduce volatilization of the carbonate coating and also to minimize oxidation of nitrite to nitrate which can occur when ozone is present (Allegrini et al. 1987; Perrino et al. 1990; Tang et al. 2009). In addition, the average denuder capture efficiency for HNO_3_ was 83.2 % in the first denuder as noted earlier, indicating little evidence of significant NO_2_ or PAN capture (Tang et al. 2009). Nevertheless, measured HNO_3_ concentrations may be overestimated to some extent at the urban CAU site but could reflect actual levels at suburban and rural sites. In future work, HNO_3_ should be selectively removed from the sampling air by first using a sodium fluoride or sodium fluoride-coated denuder as widely used in previous studies (Allegrini et al. 1987; Spataro et al. 2013). For NH_3_ sampling, Perrino and Gherardi (1999) have highlighted that in the case of citric acid, about 8 % of the collected ammonia was released after 2 h and more than 40 % after 12 h. In contrast, phosphorous acid is a suitable coating layer for a denuder line intended to determine gaseous ammonia in the atmosphere (Perrino and Gherardi 1999). However, an intercomparison study conducted by Tang et al. (2009) showed that the 14-day mean NH_3_ concentration from citric acid-coated denuders of a DELTA system was about 9 % lower than that from H_3_PO_3_-coated denuders of an Annular Denuder System. Given this, together with an overall 82.7 % NH_3_ capture efficiency as noted earlier, NH_3_ concentrations sampled at the five sites should be reasonable and acceptable, albeit with some degree of underestimation.

NO_2_ samples were collected by Gradko diffusion tubes (Gradko International Limited, UK). Each sampler consists of a 71.0-mm long × 11.0-mm internal diameter acrylic tube with colored and white thermoplastic rubber caps. Three NO_2_ samplers at each site were exposed under a PVC shelter which protected the samplers from precipitation and direct sunshine. The NO_2_ was absorbed into a 20 % triethanolamine/deionized water solution coated onto two stainless steel wire meshes within the colored cap. As indicated by the manufacturer (Gradko International Ltd, UK), the uptake rate of the tube is 68.8 × 10^−6^ m^−3^ h^−1^, the desorption efficiency is 0.98, the limit of detection is 1.6 μg NO_2_ m^−3^ over a 2-week exposure period, and the analytical expanded measurement uncertainty is ±10 %. Over the entire period, the standard deviations of each sampling across all sites ranged from 0.01 to 2.9 μg NO_2_ m^−3^ and averaged 0.8 μg NO_2_ m^−3^ (95 % confidence interval 0.7–0.9).

All the samplers were exposed for 1 month at each site and returned to the laboratory for analysis. In the laboratory, all the exposed samples were stored at 4 °C and analyzed at 1-month intervals. The HNO_3_ denuders were extracted with 10 mL 0.05 % H_2_O_2_ solution. The NH_3_ denuders were extracted with 10 mL high-purity water. Ammonium and nitrate in the extracted solutions were measured with an AA3 continuous-flow analyzer (Bran + Luebbe GmbH, Norderstedt, Germany). The detection limits were determined as 0.01 mg N L^−1^ for NH_4_^+^ and NO_3_^−^. The meshes from the NO_2_ diffusion tubes were extracted with a solution containing sulfanilamide, H_3_PO_4_, and N-1-naphthylethylene-diamine, and the NO_2_ content in the extract determined using a colorimetric method by absorption at a wavelength of 542 nm. The detection limit for NO_2_ was 0.01 mg N L^−1^. The laboratory and field blank samples were extracted and analyzed using the same methods as the exposed samples. After correcting for the corresponding blanks, the results were used for the calculation of concentrations for all measured gases.

Samples of PM_2.5_ were collected by medium-volume samplers (TH-150CIII, 100 L min^−1^, Tianhong Co., Wuhan, China) onto 90-mm quartz fiber filters (Whatman QM/A, Maidstone, UK) at all sites except ZZ because only four particle samplers were available. The quartz fiber filters were baked at 500 °C for 4 h prior to sampling to remove contaminants. The PM_2.5_ samples were collected on a 24 hourly basis from 08:00 hours to 08:00 hours the next day. More than 25 valid samples were obtained for most seasons during the sampling period at each site. Owing to precipitation or occasional sampler failure, a number of seasons have less than 20 samples.

Before and after sampling, the filters were equilibrated for 24 h in a desiccator at 25 °C and 40 ± 5 % relative humidity and then weighed with a microbalance (Sartorius, precision 10 μg). The PM_2.5_ concentrations were calculated by weight differences divided by sampling air flows. A quarter of each filter was put into a 50-mL beaker with 10 mL of high-purity water (18.2 MΩ resistivity). After a 30-min ultrasonic extraction, the extracts were filtered using 0.22-μm syringe filters, and the filtrates were stored in clean tubes at 4 °C until analyzed within 1 month of extraction. The cations (NH_4_^+^, Na^+^, Ca^2+^, K^+^, Mg^2+^) and anions (NO_3_^−^, SO_4_^2−^, F^−^, Cl^−^) in the filtrates were determined by Dionex-600 and Dionex-2100 Ion Chromatograph (Dionex Inc., Sunnyvale, CA, USA), respectively. Details of the instruments and detection limits have been provided elsewhere (Zhang et al. 2011a; Tao et al. 2014). Field blank measurements were made each month or each season at all sites.

### Meteorological data

Hourly wind speed (WS), temperature (*T*), relative humidity (RH), and daily precipitation for each site for 2011–2014 were taken from Weather Underground (http://www.underground.com/). The monthly and annual WS, *T*, RH and precipitation are respectively displayed in Fig. S1 and Table S1 in the Supplementary Information (SI). Year-to-year variations in all meteorological parameters were not significant at each site (all *p* > 0.05) except RH for CAU, SZ, and QZ.

### Statistical analyses

One-way analysis of variance (ANOVA) and paired-sample *t* tests were used to decide the significance of the differences in annual average gas (i.e., NH_3_, NO_2_, and HNO_3_) concentrations and annual average meteorological data among sites or years, as well as daily average PM_2.5_ concentrations among seasons at each site. Pearson correlation and linear regression analyses were conducted for the water-soluble inorganic ions in PM_2.5_. All statistical analyses were performed using SPSS 11.5 (SPSS Inc., Chicago, IL, USA), and significance was defined as *p* < 0.05.

## Results and discussion

### Spatial and annual variations of NH_3_, NO_2_, and HNO_3_

**Fig. 2:**
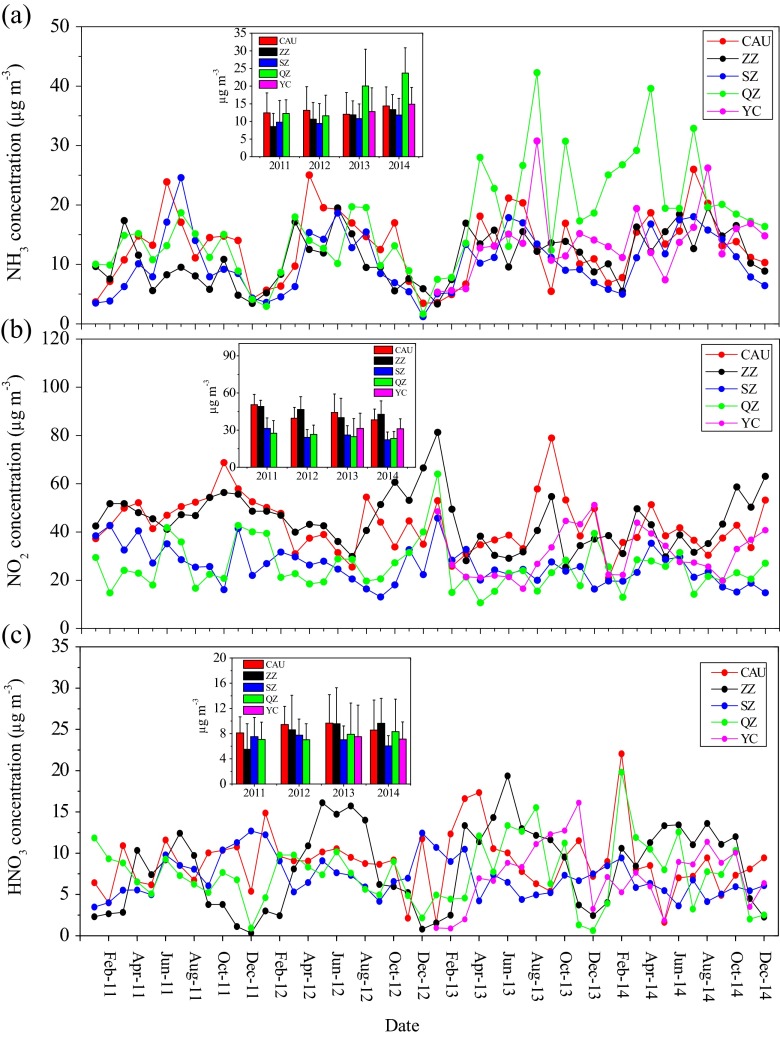
Time series of monthly average concentrations of **a** NH_3_, **b** NO_2_, and **c** HNO_3_ at the five sampling sites during 2011–2014. The annual average concentrations for the gases are shown in the *boxes inside the corresponding figures*

Monthly mean concentrations of NH_3_, NO_2_, and HNO_3_ at the five sites are shown in Fig. 2. The concentrations of NH_3_, NO_2_, and HNO_3_ across all sites were in the ranges of 1.2–42.3, 10.6–81.3, and 0.3–22.1 μg m^−3^, respectively. Their concentrations varied greatly across sites for all measured gases. The annual mean concentrations of NH_3_, NO_2_, and HNO_3_ at the five sites for the years between 2011 and 2014 are also presented in Fig. 2. The annual NH_3_ concentrations varied from 8.5 ± 3.7 μg m^−3^ at ZZ in 2011 to 23.7 ± 7.2 μg m^−3^ at QZ in 2014 (Fig. 2a). The year-to-year variations in annual concentrations of NH_3_ were sometimes significant at all sites except CAU and YC (details are given in Table S2). However, it is important to note that annual NH_3_ levels show a slight increasing trend at the five sites. This finding is consistent with the increasing trend of NH_3_ emissions during recent years in the NCP due to intensified agricultural activities (Zhang et al. 2010, 2011b). The largest annual mean NH_3_ concentration was observed at QZ (16.9 ± 5.9 μg m^−3^), followed by YC (13.8 ± 1.5 μg m^−3^), CAU (13.1 ± 1.0 μg m^−3^), ZZ (11.1 ± 2.0 μg m^−3^), and SZ (10.5 ± 1.1 μg m^−3^) (Fig. S2). This is likely due to the fact that QZ is a typical agricultural rural site with excessive N fertilizer input (about 500–600 kg N ha^−1^ year^−1^) over a large amount of agricultural land (75 % of the total land), which is the main source of NH_3_ (Clarisse et al. 2009). However, the difference in the annual NH_3_ concentrations during 2011–2014 across the five sites was not significant. High NH_3_ concentration in urban areas is associated with NH_3_ emissions from biological sources, such as humans, sewage systems, and garbage containers (Reche et al. 2002). NH_3_ is a secondary pollutant in gasoline vehicle emissions that results from the reaction which occurs in the catalytic converter between NO and H (Moeckli et al. 1996). Between 2006 and 2013, the number of civil vehicles increased from 2.39 to 5.17 million in Beijing and from 0.46 to 1.72 million in Zhengzhou (CSY 2007–2014), which could result in elevated NH_3_ emissions. In addition, large cities in China (e.g., Beijing and Zhengzhou) can receive large amount of agricultural NH_3_ from the suburban areas (Gu et al. 2014; Xu et al. 2014). In the present study, annual NH_3_ concentrations at the five sites were 4~11 times higher than the annual background atmospheric NH_3_ in North China (ca. 2.1 μg m^−3^) reported by Meng et al. (2010). NH_3_ levels at different urban, suburban, and rural sites in the world are listed in Table S3. The average concentrations of NH_3_ measured at the rural and urban sites in this study were far higher than those reported in southern China (e.g., Yang et al. 2010; Shen et al. 2013) and in other countries (e.g., Walker et al. 2004; Trebs et al. 2006; Endo et al. 2011) but were comparable to previous measurements in the NCP (e.g., Meng et al. 2011; Luo et al. 2013). NH_3_ concentrations at the suburban site (SZ) were close to those observed at suburban sites with serious NH_3_ pollution worldwide (Singh et al. 2001; Alebic-Juretic 2008; Cao et al. 2009). Our findings suggest that the NCP is still experiencing serious NH_3_ pollution in present-day China, which is closely related to the high NH_3_ emissions from N fertilizer application, intensive livestock production facilities, and high population density. For example, typical application rates of N fertilizer are 500–600 kg N ha^−1^ year^−1^ for high yields of maize and wheat in rural and suburb areas. However, less than 30 % of the N fertilizer applied is taken up by the crops and more than 20 % (ca. 100 kg N ha^−1^ year^−1^) is lost by NH_3_ emissions (Pan et al. 2012). This makes a significant contribution to high NH_3_ concentrations in the whole region. Moreover, North China is witnessing a rapid increase in livestock production facilities in suburban areas. Populations of the main livestock (pig and cattle) have increased at an annual rate of 2 % from 1996 to 2013 in North China (CSY 1997–2014). This will also result in large emissions of NH_3_.

The annual average NO_2_ concentrations ranged from 22.2 ± 6.2 μg m^−3^ at SZ in the suburb of Beijing in 2014 to 50.5 ± 8.3 μg m^−3^ at CAU in the city area in 2011 (Fig. 2b). The annual concentrations at each site varied to a different extent among the years and overall exhibited a decreasing trend during the period 2011–2014 (Fig. 2b and Table S2). This finding is in accordance with the modeling results of Wang et al. (2014b) who calculated that ambient NO_2_ concentration will decrease by 8 % during the 12th Five-Year Plan period (2011–2015) as a consequence of national NO_*x*_ control policies (e.g., new emission standards for power plants and vehicles). It is interesting to observe that the year-to-year variation exhibited the same characteristic at CAU and SZ, i.e., monthly mean values were significantly lower (*p* < 0.05) in 2014 than in 2011 but were not significantly different (*p* > 0.05) between other years (Table S2). This result suggests that NO_2_ produced in the area of the urban site can greatly affect NO_2_ concentration at the suburban site. As for inter-site comparisons, annual NO_2_ concentrations at the urban sites (CAU and ZZ, average 43.9 ± 1.0 μg m^−3^) were significantly higher (*p* < 0.05) than those at the rural and suburban sites (QZ, YC, and SZ, average 27.5 ± 3.2 μg m^−3^). Differences in annual average values between the suburban and rural sites were not significant (*p* > 0.05) (Fig. S2). It is commonly accepted that NO_2_ is a ubiquitous air pollutant in urban regions derived mainly from fossil fuel combustion processes including power plants, transportation, and industry (Streets et al. 2003). The background concentration of atmospheric NO_2_ was only about 3.7 μg m^−3^ in North China (Meng et al. 2010). In the present study, annual NO_2_ concentrations at the rural sites (23.2–31.4 μg m^−3^) were lower than those obtained in a rural area with serious NO_2_ pollution in eastern China (average 42 μg m^−3^) (Yang et al. 2010) but were much greater than those obtained in the studies of Aas et al. (2007) and Shen et al. (2013) at several rural sites in south China (Table S3), and exceed (or are close to) the annual mean NO_2_ guideline value of 30 μg m^−3^ set by the World Health Organization (WHO 2000). Annual NO_2_ concentrations at the urban sites (38.3–50.5 μg m^−3^) exceeded the WHO guideline and mostly exceeded the Chinese annual exposure limit for humans of 40 μg m^−3^ for NO_2_ (MEPC 2012). Compared to urban sites in other studies (Table S3), the urban NO_2_ concentrations in this study were similar to those obtained at most capital cities reported by Wang et al. (2014b) for the period of 2013–2014 in China and were higher than values reported for Thessaloniki, Greece (Anatolaki and Tsitouridou 2007). Combining these findings, we conclude that many large cities in China, and rural and suburban regions in the NCP, are suffering from serous NO_2_ pollution, which mainly results from high NO_*x*_ emission from the construction of new power plants and the rapid increase of vehicle numbers. According to Wang and Hao (2012), China increased its thermal power generation by 195 % and vehicle production by 300 % during 2000–2010 and NO_*x*_ emissions from power plants and transport increased by over 100 and 200 %, respectively, over the same period. The increased NO_2_ emissions from newly built large power plants in North China can even be observed by satellite (Wang et al. 2012b).

NH_3_ and NO_2_ are two primary reactive N species in air which mainly come from human activity. The monthly mean molar ratio of NH_3_ to NO_2_ were in the ranges of 0.11–1.92 at the urban sites (CAU and ZZ), 0.15–2.55 at the suburban site, and 0.11–7.38 at the rural sites (QZ and YC), with overall annual values of 0.81, 1.19, and 1.74, respectively (Fig. S3). These results indicate that the concentrations of gaseous N compounds in the air are predominantly influenced by fossil fuel combustion in urban areas and by agricultural activity in non-urban areas.

In contrast to NH_3_ and NO_2_, the annual mean concentrations of HNO_3_ at the five sites were lower and less variable, ranging from 5.5 ± 4.1 μg m^−3^ (at ZZ in 2011) to 9.7 ± 4.5 μg m^−3^ (at CAU in 2014) (Fig. 2c). The year-to-year variation in annual averages was comparatively small at each site except that ZZ and SZ showed a significant difference (*p* < 0.05) in monthly mean values between 2014 and 2011 and 2012, respectively (Table S2). Annual HNO_3_ concentrations were not significantly different (*p* > 0.05) among the five sites, with mean values of 8.9, 8.3, 7.1, 7.6, and 7.3 μg m^−3^ at CAU, ZZ, SZ, QZ, and YC, respectively (Fig. S2). This finding is not surprising because HNO_3_ is produced through many pathways in the atmosphere, including photooxidation of NO_2_ with OH, reaction of NO_3_ with VOC, hydrolysis of N_2_O_5_, and dissociation of NH_4_NO_3_ aerosol (Khoder 2002). The fate of HNO_3_ is controlled by the reaction with NH_3_, which is influenced by ambient temperature, relatively humidity, and NH_3_ concentrations (Sharma et al. 2007). Therefore, the absence of significant spatial difference of HNO_3_ in this study is likely linked to the differences among sites in the extent of oxidation of NO_2_, the contribution from other sources, and the ratio of HNO_3_ and NH_3_. For example, the correlations between monthly mean concentrations of NO_2_ and HNO_3_ were not significant at each site except for a significantly negative correlation for ZZ (Fig. S4). Moreover, NH_3_ and HNO_3_ were found to be highly positively corrected at ZZ, QZ, and YC (Fig. S5), suggesting that dissociation of NH_4_NO_3_ is the important contributor for the ambient HNO_3_. Average HNO_3_ concentrations in this study were comparable to those measured at two sites in the NCP reported by Luo et al. (2013) but much higher than those observed at three sites in south China (Shen et al. 2013) and at many sites worldwide (e.g., Endo et al. 2011; Trebs et al. 2006) (Table S3). The NCP has some of the highest air pollution in China due to the large amounts of coal combustion for industry and power plants and residential heating leading to high HNO_3_ concentrations from oxidation of NO_2_.

### Seasonal variation of gaseous NH_3_, NO_2_, and HNO_3_

**Fig. 3:**
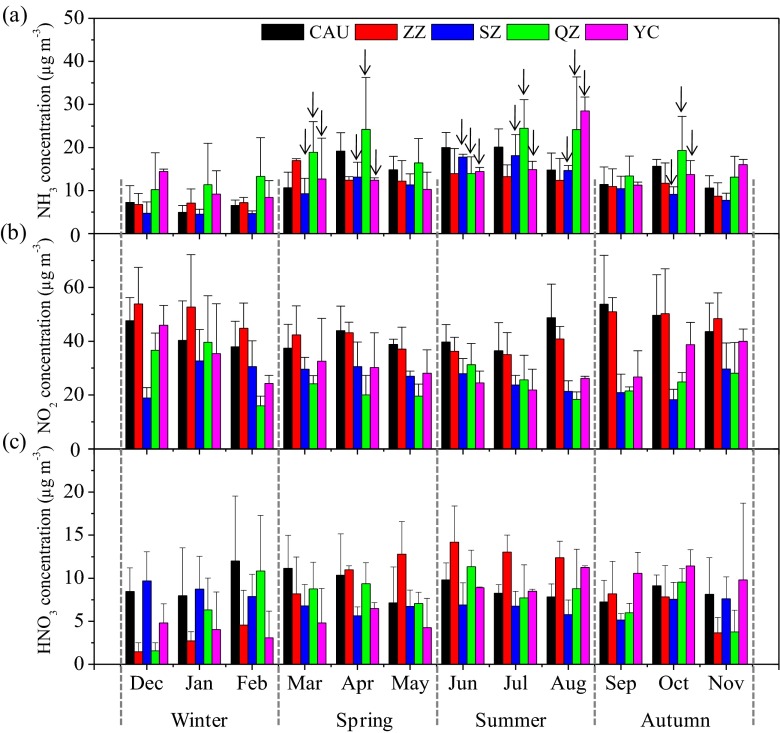
The statistics of monthly average concentrations of *a* NH_3_, *b* NO_2_, and *c* HNO_3_ during the sampling periods at the five sites. The *arrows* denote N fertilizer application for the maize-wheat crop rotation system at SZ, QZ, and YC

**Fig. 4:**
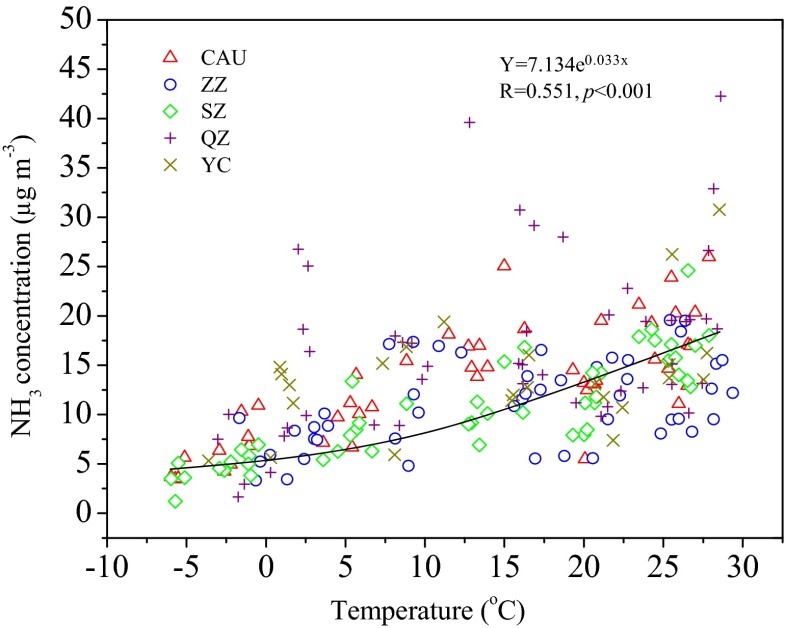
Correlation between monthly average air temperature and monthly average NH_3_ concentration across all five sites

The seasonal concentrations of NH_3_, NO_2_, and HNO_3_ are dependent on their source strength and meteorological conditions. Figure 3 shows the monthly statistics of NH_3_, NO_2_, and HNO_3_ concentrations, averaged over the 4-year period, measured at the five sites (2-year observation at YC). NH_3_ concentrations across all sites were higher in March or April, especially at the rural sites (Fig. 3(a)). This can be partly explained by the enhanced NH_3_ emission from natural and agricultural sources and city garbage, caused by the abrupt temperature increase after winter (Fig. S1a); every 5 °C temperature increase nearly doubles the volatilization potential of ammonia (Sutton et al. 2013). The highest concentrations of NH_3_ at all sites were in summer (June–August), which is due to the fact that high temperatures together with ammonium-N fertilizer use induce high NH_3_ emissions from fertilizers. As shown in Fig. 4, NH_3_ concentrations increased exponentially with the increase in air temperature at the sampling sites. The lowest concentrations of NH_3_ in winter can be ascribed to the reduced NH_3_ volatilization at low air temperature, high snow coverage, and infrequency of agricultural activities in winter (Cao et al. 2009). The highest NO_2_ concentrations at all sites were observed in autumn (September–November) or winter (December–February) with the exception of SZ, which showed comparable values between spring and winter (Fig. 3(b)). Increased NO_2_ emissions from the greater coal combustion for domestic heating (from middle November to middle March) in Northern China is the main reason for high NO_2_ concentrations in autumn/winter. Moreover, agricultural crop residues in North China are not only burned as domestic fuel but are also burned directly in the field during harvest seasons (e.g., autumn), which can also cause serious local and regional NO_2_ pollution (Duan et al. 2004). In addition, stable atmospheres and low temperatures appeared more frequently during autumn and winter (Fig. S1a, b), which are unfavorable meteorological conditions for air pollution dilution and dispersion (Chai et al. 2014). The lowest NO_2_ concentrations were observed in summer at ZZ, SZ, and YC; in spring at QZ; and were comparable between spring and summer at CAU. In summer, stronger atmospheric mixing leads to a deeper boundary layer and a dilution of pollutants emitted from the surface, and the increased photochemistry increases the oxidation of NO_2_ and its conversion rate to nitrate by reaction with OH (Yang et al. 2010). Consequently, NO_2_ concentrations were lowest in summer at most sites. In contrast, the relatively high NO_2_ concentration in summer at QZ and CAU is probably due to high NO_2_ emissions from road traffic. The seasonal pattern of HNO_3_ changes somewhat across the five sites, with the highest HNO_3_ concentrations observed in winter at CAU and SZ, in summer at QZ and ZZ, and in autumn at YC (Fig. 3(c)). Different seasonal patterns of atmospheric HNO_3_ in China were also reported in previous studies (Li et al. 2013; Luo et al. 2013; Shen et al. 2013).

### Mass concentrations of PM_2.5_ and water-soluble ions

Table 2 presents the summary statistics for daily average PM_2.5_ concentrations during the sampling periods at the four sites. The concentrations of PM_2.5_ were in the range 11.8–621.0, 19.8–692.9, 23.9–754.5, and 27.9–455.0 μg m^−3^ at CAU, SZ, QZ, and YC, respectively (data for each season per site during the sampling period are provided in Table S4). Daily average PM_2.5_ concentrations were not significantly different between the sites with the exception of significantly higher PM_2.5_ concentrations at CAU than at SZ. The average PM_2.5_ concentration at the urban site (CAU, 159.4 μg m^−3^) was comparable to the annual mean value of 123.5 μg m^−3^ in 2009/2010 in the urban area of Beijing (Zhao et al. 2013). Also, the average daily PM_2.5_ concentration at the suburban (SZ, 141.5 μg m^−3^) and rural (153.9 μg m^−3^ at QZ and 141.8 μg m^−3^ at YC) sites was similar to those obtained at sites with corresponding land-use types in the NCP (Shen et al. 2011). The daily average PM_2.5_ concentration was a factor of 2.1 (95 % confidence interval 1.99–2.25), 1.9 (1.72–2.04), 2.1 (1.89–2.21), and 1.9 (1.75–2.03) greater than the Chinese Grade II standard for daily PM_2.5_ concentration (75 μg m^−3^, MEPC 2012) at CAU, SZ, QZ, and YC, respectively. When compared to the WHO guideline for daily PM_2.5_ concentration (25 μg m^−3^, WHO 2005), the ratios were even higher, being 6.4 (5.99–6.76) at CAU, 5.7 (5.18–6.14) at SZ, 6.2 (5.67–6.64) at QZ, and 5.7 (5.24–6.11) at YC. More than 70 % of the sampling days had daily average PM_2.5_ concentration above the Chinese Grade II standard at the four sites, especially at YC (94 %). Compared with the WHO standard for daily average PM_2.5_, almost all (>98 %) of the daily PM_2.5_ concentration exceeded the standard. Obviously, severe PM_2.5_ pollution not only existed in the urban area but also in suburban and rural areas in the NCP.

**Table 2:** Summary statistics for daily average PM_2.5_ concentrations (μg m^−3^) during the sampling period at the four sites

	CAU	SZ	QZ	YC
Mean	159.4	141.5	153.8	141.8
Median	137.3	110.3	126.4	127.7
Min	11.8	19.8	23.9	27.9
Max	621.0	692.9	754.5	455.0
SD	95.8	105.9	101.5	68.4
*N*	384	299	270	155
ECGS (%)^a^	80.5	72.6	79.6	93.5
EWHOS (%)^b^	98.2	99.7	99.6	100

*N* number of samples

^a^The proportion of sampling days which had concentrations of PM_2.5_ exceeded the Chinese Grade II standard

^b^The proportion of sampling days which had concentrations of PM_2.5_ exceeded the WHO standard

**Fig. 5:**
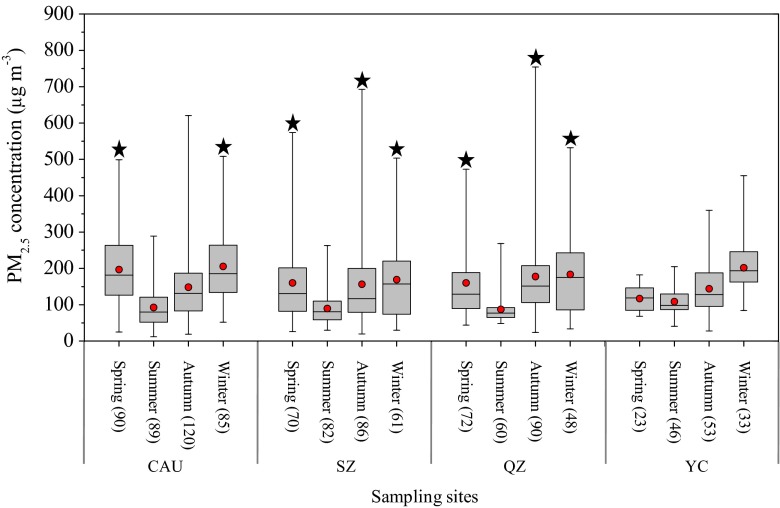
Seasonal PM_2.5_ concentrations at the four sites. The *box and whiskers* denote the minimum, 25th percentile, median, 75th percentile, and maximum; the *dots* denote the mean values, and those without statistical significance (*p* > 0.05) among them are marked with a *star*

At four sites, the daily PM_2.5_ concentrations during summer were lower than those in other seasons (Fig. 5). Higher rainfall in summer at all sites (Fig. S1d) promotes the scavenging of particles by wet deposition. In addition, higher temperatures during summer (Fig. S1a) favor the volatilization of fine particle nitrate to NH_3_ and HNO_3_ (Seinfeld and Pandis 2006). Different seasonal characteristics for highest PM_2.5_ concentrations were found in the present study. At CAU, the maximum concentrations were in spring and winter, with no significant difference between the two seasons. This seasonal pattern is consistent with that for the period 2005–2008 in Beijing investigated by Yu et al. (2011) but is different from the finding of Zhao et al. (2013) who reported similar seasonal PM_2.5_ concentrations across seasons in Beijing in 2009/2010, ascribed to the promotion of electricity and natural gas use. So, our result may imply that combustion of fossil fuel is still the important source of PM_2.5_ in Beijing, regardless of differences in meteorological conditions (e.g., wind direction, wind speed) during experiment periods between the two studies. At QZ and SZ, the concentrations were not significantly different between spring, autumn, and winter. As revealed by Yu et al. (2011), high PM_2.5_ concentrations in spring in Beijing were mainly dominated by geogenic particles from the west and northwest of China via atmospheric transport. In contrast, high concentrations of PM_2.5_ in winter and autumn resulted from the combination of coal and biomass burning for domestic home heating and direct burning of agricultural residues in the field (Hu et al. 2014). Moreover, stable meteorological conditions during autumn and winter (see “Seasonal variation of gaseous NH_3_, NO_2_, and HNO_3_”) also lead to the accumulation of air pollutants. The PM_2.5_ concentrations at YC were significantly higher only in winter as compared to the other three seasons, among which there was no significant difference in PM_2.5_ concentration. Low PM_2.5_ concentration in spring at YC is associated with a combination of fewer samples collected in the spring of 2013 (Table S4) and missing days with serious particle pollution.

The average concentrations of water-soluble ionic species during the sampling period at the four sites are presented in Table 3. The proportion of the water-soluble ions in PM_2.5_ was similar for the urban site (36 %), the suburban site (34 %), and the rural sites (average 40 %). The concentrations of ions at the urban and suburban sites were both in the order NH_4_^+^ > Ca^2+^ > K^+^ > Na^+^ > Mg^2+^ for the cations and NO_3_^−^ > SO_4_^2−^ > Cl^−^ > F^−^ for the anions. At the rural sites, the concentration order was NH_4_^+^ > K^+^ > Ca^2+^ > Na^+^ > Mg^2+^ for the cations and SO_4_^2−^ > NO_3_^−^ > Cl^−^ > F^−^ for the anions. The SO_4_^2−^, NO_3_^−^, and NH_4_^+^ are the dominant ionic species, contributing 29–39 % of the average PM_2.5_ mass across the four sites (Table 3). The sampling sites in the present study were located in Beijing and its neighboring provinces (far from the ocean), where the contribution to aerosols from sea salt spray could be ignored (Yuan et al. 2004). The inconsistent order of Ca^2+^ and K^+^ between non-rural and rural sites is likely due to the differences in contributions from road and soil dust and biomass burning, as the fine mode Ca^2+^ and K^+^ are widely regarded as indicators of mineral dust and biomass burning, respectively (Zhao et al. 2010). The average mass ratios of NO_3_^−^/SO_4_^2−^ were 1.15 ± 0.90, 1.10 ± 0.82, 0.81 ± 0.53, and 0.81 ± 0.49 for CAU, SZ, QZ, and YC, respectively. The higher ratios at CAU and SZ indicate a greater fraction of particles sourced from automobile exhaust. Lower NO_3_^−^/SO_4_^2−^ ratio at QZ and YC could reflect the dominant coal combustion sources for particles.

**Table 3:** Average mass concentrations of PM_2.5_ species during the sampling period at the four sites

	CAU	SZ	QZ	YC
	Mean ± SD	Mean ± SD	Mean ± SD	Mean ± SD
PM_2.5_ (μg m^−3^)	159.40 ± 95.76	141.50 ± 105.89	153.85 ± 101.53	141.78 ± 68.38
NO_3_^−^ (μg m^−3^)	19.23 ± 18.98	16.26 ± 19.28	16.01 ± 15.95	18.09 ± 15.13
SO_4_^2−^ (μg m^−3^)	18.61 ± 17.92	15.21 ± 14.80	20.08 ± 16.06	24.52 ± 14.66
NH_4_^+^ (μg m^−3^)	10.78 ± 10.26	9.67 ± 9.54	10.49 ± 8.31	12.25 ± 7.02
Cl^−^ (μg m^−3^)	3.74 ± 4.82	2.28 ± 2.70	3.52 ± 4.36	2.42 ± 2.81
Ca^2+^ (μg m^−3^)	2.64 ± 1.98	1.82 ± 1.72	1.60 ± 1.59	1.25 ± 1.06
Na^+^ (μg m^−3^)	0.93 ± 0.87	0.84 ± 0.73	0.71 ± 0.69	0.70 ± 0.69
K^+^ (μg m^−3^)	1.32 ± 1.24	1.21 ± 1.16	1.73 ± 1.24	2.04 ± 1.43
Mg^2+^ (μg m^−3^)	0.32 ± 0.27	0.26 ± 0.18	0.23 ± 0.23	0.24 ± 0.26
F^−^ (μg m^−3^)	0.26 ± 0.26	0.18 ± 0.19	0.21 ± 0.24	0.27 ± 0.26
Sum of ionic species (μg m^−3^)	58.84 ± 48.88	47.73 ± 43.78	55.31 ± 41.95	61.97 ± 35.63
Secondary inorganic aerosol (μg m^−3^)	48.6 ± 44.9	41.2 ± 40.8	46.6 ± 37.2	54.9 ± 33.1
WSII (%)^a^	0.35 ± 0.18	0.34 ± 0.17	0.35 ± 0.15	0.44 ± 0.15
SIA (%)^b^	0.29 ± 0.17	0.29 ± 0.16	0.30 ± 0.14	0.39 ± 0.15

^a^Proportion of water-soluble inorganic ions in PM_2.5_

^b^Proportion of secondary inorganic ions in PM_2.5_

**Fig. 6:**
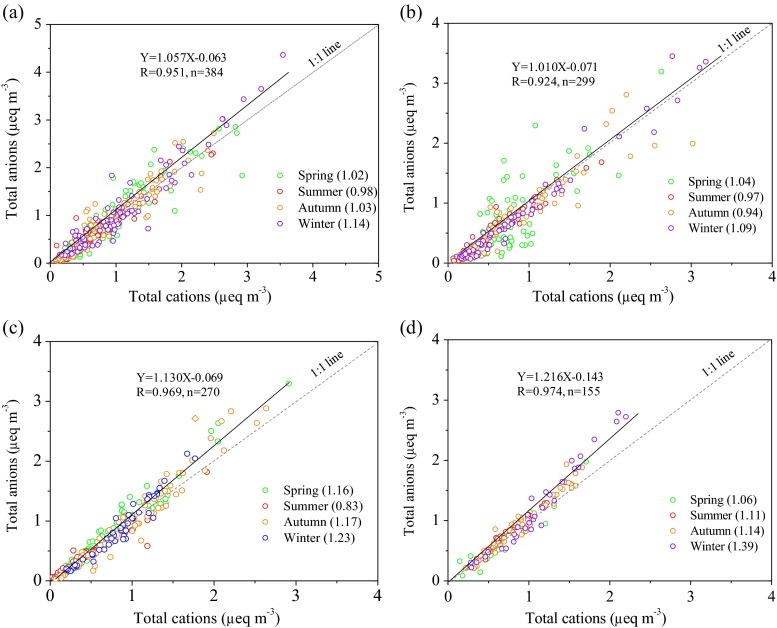
The molar inorganic ion balance in PM_2.5_ at the four sites: **a** CAU, **b** SZ, **c** QZ, and **d** YC

Figure 6 illustrates the acid-base balance of the inorganic ions in PM_2.5_ at the four sites. The ion balance expresses the equivalent concentration (μeq m^−3^) of total inorganic anions (sum of NO_3_^−^, SO_4_^2−^, Cl^−^, F^−^) to cations (sum of NH_4_^+^, Ca^2+^, K^+^, Na^+^, and Mg^2+^). The correlation coefficients for the anion versus cation concentration data stratified by season all were greater than 0.92 at all sites, suggesting a common origin of the ions in PM_2.5_. The slopes (anion/cation) of the linear regressions for all PM_2.5_ samples were equal to the theoretical equivalent ratio of 1 at CAU (1.06) and SZ (1.01) but were greater than 1.1 at QZ (1.13) and YC (1.22). These results imply that the aerosols were neutral at the urban and suburban sites but acidic at the rural sites. The acidic PM_2.5_ observed at the rural sites is most likely due to relatively low concentrations of Ca^2+^ which play an important role in spatial distribution of PM_2.5_ acidity (He et al. 2012). Fully neutralized aerosol have been widely observed in different areas worldwide (Shon et al. 2012; Tao et al. 2014), but acidic aerosol has also been reported by many previous studies (Wang et al. 2006; Zhang et al. 2011a; He et al. 2012). The seasonal anion/cation ratios at the four sites, i.e., the slopes of linear regressions for the seasonally stratified data, were found to vary moderately (Fig. 6). This characteristic is consistent with the findings of Shon et al. (2012), who suggested that seasonal variation in ratio of anion/cation was caused by unmeasured cations such as ferric and non-ferric components.

### Secondary inorganic aerosol

**Fig. 7:**
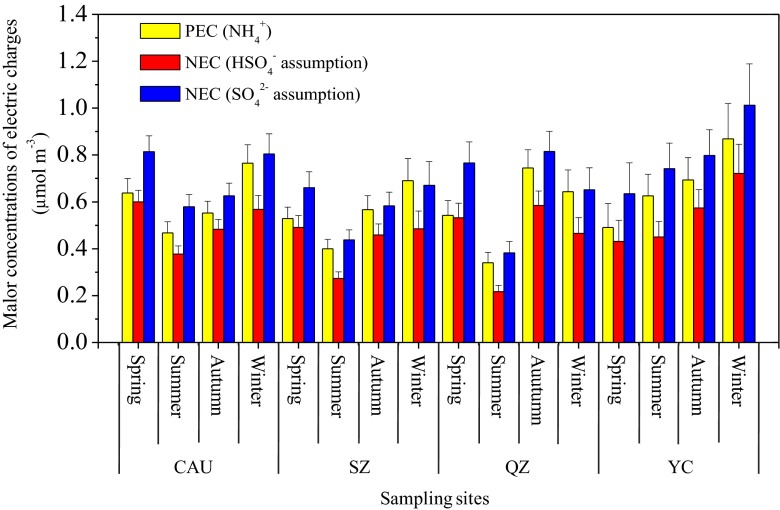
Molar concentrations of positive electric charges of NH_4_^+^ (PEC) and negative electric charges of NO_3_^−^ and SO_4_^2−^ (NEC)

Ammonia in the atmosphere can react with H_2_SO_4_ to form ammonium sulfate ((NH_4_)_2_SO_4_) and ammonium bisulfate (NH_4_HSO_4_) and react with HNO_3_ and HCl to form ammonium nitrate (NH_4_NO_3_) and ammonium chloride (NH_4_Cl) (Ianniello et al. 2010). These compounds are referred to as “secondary inorganic aerosol (SIA)” in this paper. The 4Pearson correlation coefficients between the molar concentrations of NO_3_^−^, SO_4_^2−^, and Cl^−^ in PM_2.5_ are presented in Table S5. At the four sites, the correlation coefficients (CCs) between NH_4_^+^, SO_4_^2−^, and NO_3_^−^ were comparable, but both of them were higher than CCs between NH_4_^+^ and Cl^−^. Moreover, the CCs between NH_4_^+^ and the sum of NO_3_^−^ and SO_4_^2−^ at all sites (except SZ) were higher than those between NH_4_^+^ and the sum of NO_3_^−^, SO_4_^2−^, and Cl^−^. These results mean NH_4_^+^ was probably mainly combined with NO_3_^−^ and SO_4_^2−^. In order to further understand the neutralization processes between them, we calculated the molar concentrations of positive electric charges of NH_4_^+^ (PEC = NH_4_^+^/18) and negative electric charges of NO_3_^−^ and SO_4_^2−^ (NEC = NO_3_^−^ / 62 + 2 × SO_4_^2−^ / 96). If all sulfate was assumed to be in the form of HSO_4_^−^, then NEC = (NO_3_^−^ / 62 + SO_4_^2−^ / 96) (Louie et al. 2005; Zhao et al. 2013). The seasonal average PEC and NEC are shown in Fig. 7. At all sites, we found that NH_4_^+^ was enough to match NO_3_^−^ and SO_4_^2−^ to form NH_4_HSO_4_ in all four seasons and not sufficient to meet the complete neutralization of SO_4_^2−^ and NO_3_^−^for formation of (NH_4_)_2_SO_4_ aerosol in most seasons. This indicates acid-rich conditions at the study sites. Interestingly, our findings at CAU differ from results for urban sites in Beijing and its surrounding provinces during 2009–2010 when NH_4_^+^ concentrations were far from enough to match NO_3_^−^ and SO_4_^2−^ throughout the year (Zhao et al. 2013). We can infer the enhanced alkalization of the atmosphere in Beijing and/or its surrounding areas because the levels of NO_3_^−^ and SO_4_^2−^ were closely comparable between the two studies. The average molar ratio of NH_4_^+^ to SO_4_^2−^ were 3.07 ± 2.08 at CAU, 3.96 ± 2.53 at SZ, 2.84 ± 1.23 at QZ, and 2.85 ± 0.97 at YC, suggesting the main form of (NH_4_)_2_SO_4_. According to Guo et al. (2014), reductions in emissions of the aerosol precursor gases from transportation and industry are essential to mediate severe haze pollution in China. Based on our findings, we suggest that a feasible and ideal pathway to control PM_2.5_ pollution in the NCP should target ammonia and acid gases together.

The average SIA concentrations (the sum of NO_3_^−^, SO_4_^2−^, and NH_4_^+^) were 48.6 ± 44.9, 41.2 ± 40.8, 46.6 ± 37.2, and 54.9 ± 33.1 μg m^−3^ at CAU, SZ, QZ, and YC, respectively (Table 3). All averages exceeded the Chinese ambient air quality standard for annual average value of PM_2.5_ (grade II, 35 μg m^−3^) (MEPC 2012), suggesting serious SIA pollution at all sites. The SIA concentrations in the present study were much higher than those reported in many European countries, the USA, and other developed countries (Shen et al. 2013), as well as many other cities in China (Zhang et al. 2011a). SIA concentrations at CAU were comparable to values in urban Beijing reported by a recent study (Zhao et al. 2013) but were obviously higher than 2001–2003 observations at five urban sites in Beijing (average 35.9 μg m^−3^, Wang et al. 2005). This reflects enhanced emissions of the gaseous precursors (i.e., NH_3_, SO_2_, and NO_*x*_) as a result of substantial increase in vehicle traffic, coal consumption, etc.

**Fig. 8:**
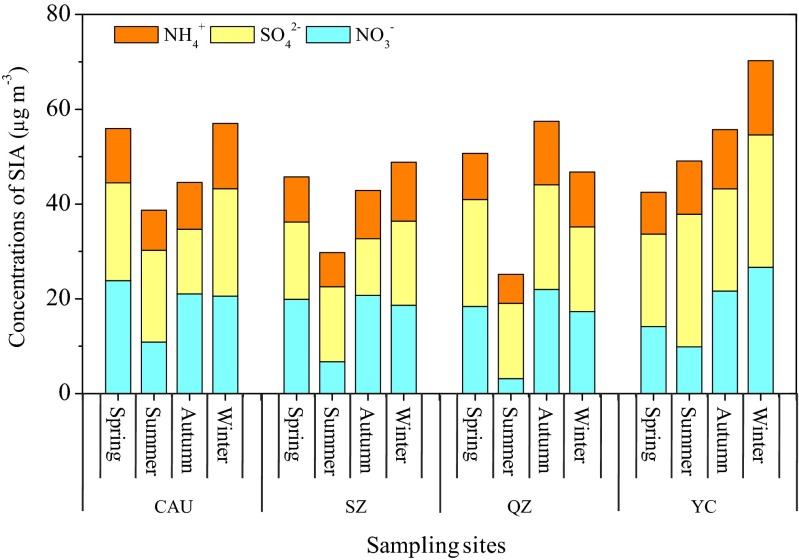
Average seasonal concentrations of secondary inorganic aerosol at the four sites

Seasonal concentrations of SIA at the four sites are shown in Fig. 8. At all sites except YC, the seasonal pattern of SIA is similar to that of PM_2.5_ (Fig. 5), consistent with the findings of Yin et al. (2014). The SO_4_^2−^ concentrations at the urban and suburban sites (CAU and SZ) exhibited a consistent seasonal variation, with the order ranked by winter > spring > summer > autumn (Fig. 8). It should be noted that the average SO_4_^2−^ concentration in winter (22.7 μg m^−3^) at CAU was slightly higher than the concentration (19.1 μg m^−3^) observed in Beijing for winter in 2009 (Zhao et al. 2013). The similar level of sulfate loading suggests that the effect of gas desulfurization in power plants might be greatly offset by the increasing coal consumption. In contrast, the seasonal SO_4_^2−^ concentrations at the rural sites were ranked in different orders: spring, autumn > winter > summer at QZ and summer, winter > autumn > spring at YC. We observed relatively high SO_4_^2−^ concentrations in summer at each site. However, SO_2_ concentrations were usually lowest in summer (Table S6) not only because of lower coal combustion but also owing to the increased photochemical oxidation activity, which was one of the important factors for the enhanced sulfate level in summer (Husain and Dutkiewicz 1990). At all sites, NO_3_^−^ concentrations were distinctly lower in summer than in the other three seasons (Fig. 8). Nitrate is more sensitive to temperature, and higher temperature in summer does not favor the formation of nitrate. Moreover, a large portion of ammonium nitrate (NH_4_NO_3_) could evaporate from the filters, especially in summer (Ianniello et al. 2011). In contrast, low temperature and high emissions of NO_*x*_ were favorable for formation of NO_3_^−^ aerosol and the reaction with NH_4_^+^ (Mariani and Mello 2007). As already discussed in “Seasonal variation of gaseous NH_3_, NO_2_, and HNO_3_,” NO_*x*_ emissions increase between mid-September and mid-March which, in combination with winter heating under relatively low temperature during that period (Fig. S1a), lead to high NO_3_^−^ concentrations. NH_4_^+^ concentrations at the four sites were higher in autumn and winter than in spring and summer. The formation of NH_4_^+^ depends on air concentrations of acid gases, temperature, water availability (Khoder 2002), as well as flux rates of NH_3_ (Nemitz et al. 2001). Compared with spring and summer, the lower temperature and higher SO_2_ and NO_*x*_ emissions in winter and autumn, especially in winter, favor the gas-to-particle phase conversion and result in higher NH_4_^+^ aerosol concentration. Previous studies also showed higher NH_4_^+^ in winter compared with higher NH_3_ in summer (Shen et al. 2009; Zhang et al. 2011a; Li et al. 2012).

## Summary and conclusions

This study provides insights into the characteristics of variations in atmospheric pollutants over three typical land-use types in the North China Plain during China’s 12th FYP (2011–2015) period, which targeted the reduction of national NO_*x*_ emissions, as well as SO_2_ and primary particles. The major results and conclusions are as follows:

1. Atmospheric NH_3_ concentrations showed clear spatial variation among the five sites. However, it was found that the difference in annual NH_3_ concentrations was not significant across all sites. High NH_3_ concentration observed at the urban site was probably due to high emissions from biological sources (e.g., sewage systems and garbage containers) and vehicles in the urban area, as well as agricultural activity in the suburban area. Annual average NH_3_ concentrations showed consistent increasing trends at the five sites, reflecting the elevated NH_3_ emission intensities from transportation, agriculture, and livestock husbandry.
2. Annual average NO_2_ concentrations exhibited obvious spatial difference, showing significantly higher concentrations at the urban site than at the suburban and rural sites. An overall decreasing trend of annual NO_2_ concentrations was observed at all sites, likely related to implementation of the national controls on NO_*x*_ emissions. All annual averages, however, exceeded (or were close to) the Chinese annual NO_2_ exposure limit for humans, indicating serious atmospheric NO_2_ pollution not only at the urban site but also at suburban and rural sites resulting from local emission sources and atmospheric transport.
3. Unlike for NH_3_ and NO_2_, annual average HNO_3_ concentrations were relatively low and showed small spatial and annual variations.
4. The PM_2.5_ pollution was severe in the NCP, with more than 70 % of sampling days across the sites exceeding the Chinese Grade II standard for daily PM_2.5_ concentration. Ion balance calculations indicated that PM_2.5_ was neutral at the urban and suburban sites and acidic at the rural sites.
5. NO_3_^−^, SO_4_^2−^, and NH_4_^+^ were the dominant ionic particulate species at the four sites and accounted for 29–39 % of the PM_2.5_ mass. NH_4_^+^ was the dominant cation at all sites, whereas NO_3_^−^ and SO_4_^2−^ were the dominant anions at the non-rural and rural sites, respectively. NH_4_^+^ was insufficient to fully neutralize SO_4_^2−^ and NO_3_^−^ at all sites, indicating an acid-rich condition. The seasonal variation of SIA was similar to that of PM_2.5_, implying that a reduction of the concentrations of SIA is a feasible way to control PM_2.5_ pollution in the NCP, by directly targeting ammonia or/and acid gases. Compared with observations of the three dominant ions in Beijing in previous studies, enhanced alkalization of the atmosphere was found.
6. Similar seasonal variations were observed for concentrations of NH_3_, NO_2_, and aerosols NO_3_^−^ and NH_4_^+^ over the three land-use types, whereas seasonal variation of PM_2.5_ and HNO_3_ concentrations showed different spatial characteristics. All above seasonal patterns were affected by meteorological condition and pollution sources.

## Electronic supplementary material

ESM 1 (DOCX 689 kb)
