# Supplementary material for: Characteristics of ammonia, acid gases, and PM2.5 for three typical land-use types in the North China Plain
Source: Environ Sci Pollut Res Int. 2015 Oct 27;23:1158–72. doi: 10.1007/s11356-015-5648-3 (PMC4713460; doi:10.1007/s11356-015-5648-3)
Supplement: Supplementary file 1 — (DOCX 689 kb) [file 11356_2015_5648_MOESM1_ESM.docx]

**Characteristics of ammonia, acid gases and PM_2.5_ for three typical land use types in the North China Plain**

Wen Xu **·** Qinghua Wu **·** Xuejun Liu **·** Aohan Tang **·** Anthony J. Dore **·** Mathew R.

Heal

W. Xu **·** Q. Wu **·** X. Liu **·** A. Tang

College of Resources and Environmental Sciences, China Agricultural University, Beijing 100193, China.

A. J. Dore

Centre for Ecology and Hydrology, Edinburgh, Bush Estate, Penicuik, Midlothian EH26 0QB, UK.

M. R. Heal

School of Chemistry, The University of Edinburgh, David Brewster Road, Edinburgh, EH9 3FJ, United Kingdom

Corresponding author: [liu310@cau.edu.cn](mailto:liu310@cau.edu.cn); Tel: +86 10 62733459; Fax: +86 1062731016

**Captions**

Figure S1. Temporal variations in monthly average temperature (a), wind speed (b), relative humidity (c), and in monthly rainfall (d) during 2011-2014 at CAU, ZZ, SZ, QZ, and YC sites in the NCP.

Figure S2. Annual mean concentrations of NH_3_, NO_2_ and HNO_3_ over the sampling period at the five sites. Different letters on bars with the same colour indicate significant difference in annual concentrations between the sites at *p*<0.05.

Figure S3. Variations in the monthly mean molar ration of NH_3_ to NO_2_ at the five sites between 2011 and 2014.

Figure S4. Correlations and linear regressions between monthly mean NO_2_ and HNO_3_ concentrations at the sampling sites: a) CAU, b) ZZ, c) SZ, d) QZ and e) YC.

Figure S5. Correlations and linear regressions between monthly mean NH_3_ and HNO_3_ concentrations at the sampling sites: a) CAU, b) ZZ, c) SZ, d) QZ and e) YC.

Table S1. Annual meteorological conditions during the 2011-2014 sampling period at the five sites.

Table S2. Ratio of annual average concentrations (μg m^-3^) of NH_3_, NO_2_ and HNO_3_, calculated from annual averages between the different years during 2011-2014 at the five sites.

Table S3. Comparison of the concentrations of NH_3_, NO_2_ and HNO_3_ (μg m^-3^) in the NCP with other areas.

Table S4. Summary statistics of daily average concentrations (μg m^-3^) of PM_2.5_ in each season from Mar. 2012 to Nov. 2014 at the four sites.

Table S5. Pearson correlation coefficients between molar concentrations of NH_4_^+^, NO_3_^-^, SO_4_^2-^ and Cl^-^ in PM_2.5_ at the four sites.

Table S6. Seasonal SO_2_ concentrations (μg m^-3^)^a^ for sampling periods at CAU, SZ and QZ

**Figure S1**. Temporal variations in monthly average temperature (a), wind speed (b), relative humidity (c), and in monthly rainfall (d) during 2011-2014 at CAU, ZZ, SZ, QZ, and YC sites in the NCP.

**Figure S2**. Annual mean concentrations of NH_3_, NO_2_ and HNO_3_ over the sampling period at the five sites. Different letters on bars with the same colour indicate significant difference in annual concentrations between the sites at *p*<0.05.

**Figure S3**. Variations in the monthly mean molar ratios of NH_3_ to NO_2_ at the five sites between 2011 and 2014.

 **Figure S4**. Correlations and linear regressions between monthly mean NO_2_ and HNO_3_ concentrations at the sampling sites: a) CAU, b) ZZ, c) SZ, d) QZ and e) YC.

**Figure S5**. Correlations and linear regressions between monthly mean NH_3_ and HNO_3_ concentrations at the sampling sites: a) CAU, b) ZZ, c) SZ, d) QZ and e) YC.

**Table S1**. Annual meteorological conditions during the 2011-2014 sampling period at the five sites.

| Site | Year | Temperature (°C) | Relative humidity (%) | Wind speed  (m s^-1^) | Precipitation  (mm) |
| --- | --- | --- | --- | --- | --- |
| CAU | 2011 | 12.2 ± 11.6a | 55.8 ± 16.7a | 2.9 ± 0.8a | 720.6 ± 83.5a |
|  | 2012 | 11.7 ± 12.3a | 55.5 ± 11.6a | 2.8 ± 0.5a | 733.2 ± 78.5a |
|  | 2013 | 12.1 ± 11.5a | 72.4 ± 16.2b | 2.6 ± 0.5a | 579.1 ± 73.1a |
|  | 2014 | 13.3 ± 11.0a | 50.8 ± 12.9a | 2.5 ± 0.5a | 405.9 ± 42.8a |
| ZZ | 2011 | 14.3 ± 10.1a | 82.5 ± 9.1a | 2.5 ± 0.3a | 637.3 ± 77.9a |
|  | 2012 | 14.6 ± 10.6a | 81.3 ± 7.0a | 2.4 ± 0.4a | 498.7 ± 54.3a |
|  | 2013 | 15.8 ± 10.5a | 82.0 ± 6.9a | 2.5 ± 0.4a | 353.2 ± 32.3a |
|  | 2014 | 15.7 ± 9.3a | 82.6 ± 10.3a | 2.5 ± 0.3a | 371.8 ± 34.2a |
| SZ | 2011 | 12.2 ± 11.6a | 55.8 ± 16.7a | 2.9 ± 0.8a | 690.5 ± 133.4a |
|  | 2012 | 11.7 ± 12.3a | 55.5 ± 11.6a | 2.8 ± 0.5a | 519.7 ± 50.2a |
|  | 2013 | 12.5 ± 11.6a | 72.8 ± 16.2b | 2.6 ± 0.4a | 462.2 ± 58.3a |
|  | 2014 | 13.3 ± 11.0a | 50.8 ± 12.9a | 2.5 ± 0.5a | 405.9 ± 42.8a |
| QZ | 2011 | 14.5 ± 10.6a | 45.7 ± 18.0a | 1.5 ± 0.3a | 432.2 ± 41.8a |
|  | 2012 | 14.3 ± 11.2a | 64.9 ± 14.9b | 1.4 ± 0.2a | 410.7 ± 54.1a |
|  | 2013 | 15.0 ± 11.0a | 50.6 ± 11.2a | 1.5 ± 0.3a | 395.7 ± 48.4a |
|  | 2014 | 15.8 ± 9.9a | 48.4 ± 14.1a | 1.4 ± 0.2a | 315.2 ± 29.4a |
| YC | 2013 | 14.1 ± 11.3a | 61.2 ± 11.0a | 1.9 ± 0.4a | 554.7 ± 78.7a |
|  | 2014 | 14.8 ± 9.9a | 53.4 ± 10.9a | 1.9 ± 0.4a | 277.7 ± 23.8a |

Values in a column at each site without the same letter indicate significant differences between the years at *p <*0 *.*05.

**Table S2**. Ratio of annual average concentrations (μg m^-3^) of NH_3_, NO_2_ and HNO_3_, calculated from annual averages between the different years during 2011-2014 at the five sites.

| Site | Pollutants | Year/year | | | | | |
| --- | --- | --- | --- | --- | --- | --- | --- |
|  |  | 2012/2011 | 2013/2011 | 2014/2011 | 2013/2012 | 2014/2012 | 2014/2013 |
| CAU | NH_3_ | 1.05 (1.09)^a^ | 0.97 (1.09) | 1.16 (0.96) | 0.92 (0.91) | 1.10 (0.80) | 1.20 (0.88) |
|  | NO_2_ | 0.78 (1.06)^*^ | 0.88 (1.80) | 0.76 (1.05)^**^ | 1.12 (1.70) | 0.97 (0.99) | 0.87 (0.58) |
|  | HNO_3_ | 1.16 (1.13) | 1.19 (1.77) | 1.05 (1.88) | 1.03 (1.57) | 0.91 (1.66) | 0.88 (1.06) |
|  |  |  |  |  |  |  |  |
| ZZ | NH_3_ | 1.25 (1.26) | 1.39 (1.06)^*^ | 1.57 (1.13)^**^ | 1.11 (0.84) | 1.26 (0.90) | 1.13 (1.07) |
|  | NO_2_ | 0.95 (2.09) | 0.82 (3.17) | 0.87 (2.20)^*^ | 0.86 (1.52) | 0.92 (1.06) | 1.07 (0.69) |
|  | HNO_3_ | 1.57 (1.35)^**^ | 1.74 (1.40)^**^ | 1.75 (0.96)^**^ | 1.11 (1.04) | 1.12 (0.72) | 1.01 (0.69) |
| SZ | NH_3_ | 0.96 (0.92) | 1.10 (0.67) | 1.21 (0.77) | 1.15 (0.73) | 1.25 (0.84)^*^ | 1.09 (1.14) |
|  | NO_2_ | 0.77 (0.73)^**^ | 0.83 (0.89) | 0.71 (0.73)^**^ | 1.07 (1.22) | 0.92 (1.00) | 0.86 (0.82) |
|  | HNO_3_ | 1.03 (0.84) | 0.93 (0.72) | 0.80 (0.54) | 0.91 (0.86) | 0.78 (0.64)^*^ | 0.86 (0.75) |
|  |  |  |  |  |  |  |  |
| QZ | NH_3_ | 0.95 (1.51) | 1.64 (2.68)^*^ | 1.93 (1.85)^**^ | 1.73 (1.78)^**^ | 2.04 (1.23)^**^ | 1.18 (0.69) |
|  | NO_2_ | 0.96 (0.74) | 0.90 (1.42) | 0.85 (0.56) | 0.94 (1.92) | 0.88 (0.75) | 0.94 (0.39) |
|  | HNO_3_ | 0.99 (0.93) | 1.11 (1.81) | 1.18 (1.88) | 1.12 (1.95) | 1.19 (2.02) | 1.05 (1.04) |
| YC | NH_3_ | n.d.^b^ | n.d. | n.d. | n.d. | n.d. | 1.16 (0.71) |
|  | NO_2_ | n.d. | n.d. | n.d. | n.d. | n.d. | 0.99 (0.65) |
|  | HNO_3_ | n.d. | n.d. | n.d. | n.d. | n.d. | 0.95 (0.54) |

^a^The values in parenthesis are ratios of standard errors of annual concentrations

^b^ No data

^*^Significant differences in monthly mean concentrations of NH_3_, NO_2_ and HNO_3_ between the different years at *p*<0.05

^**^ Significant differences in monthly mean concentrations of NH_3_, NO_2_ and HNO_3_ between the different years at *p*<0.01

**Table S3**. Comparison of the concentrations of NH_3_, NO_2_ and HNO_3_ (μg m^-3^) in the NCP with other areas.

| Location | Type | Period | Concentration |  |  | Reference |
| --- | --- | --- | --- | --- | --- | --- |
|  |  |  | NH_3_ | NO_2_ | HNO_3_ |  |
| Beijing, north China | Urban | Jan. 2011-Dec. 2014 | 13.0 ± 5.9 | 43.2 ± 11.3 | 8.9 ± 3.7 | This study |
|  | Suburban | Jan. 2011-Dec. 2014 | 10.5 ± 5.1 | 25.9 ± 7.8 | 7.1 ± 2.4 |  |
| Zhengzhou, north China | Urban | Jan. 2011-Dec. 2014 | 11.1 ± 4.4 | 44.6 ± 11.3 | 8.3 ± 5.0 |  |
| Quzhou, north China | Rural | Jan. 2011-Dec. 2014 | 16.9 ± 8.7 | 25.5 ± 9.8 | 7.6 ± 3.9 |  |
| Yucheng, north China | Rural | Jan. 2013-Dec. 2014 | 13.8 ± 5.8 | 31.2 ± 10.2 | 7.3 ± 4.0 |  |
| Beijing, north China | Urban | Feb. 2008-Jul. 2010 | 16.5 ± 3.1 | n.d. | n.d. | Meng et al. (2011) |
|  | Rural | Jan. 2007-Jul. 2010 | 5.8 ± 3.0 | n.d. | n.d. |  |
| Beijing, north China | Rural | Jun. 2006-Sep. 2008 | 17.6 | 30.6 | n.d. | Shen et al. (2009) |
| Baoding, north China | Urban | Jan. 2011-Dec.2011 | 9.4 | 39.1 | 9.9 | Luo et al. (2013) |
| Shanxi, north China | Rural | Apr. 2010-Mar. 2011 | 8.1 | 27.3 | 7.2 |  |
| Xi'an, north China | Urban | Apr. 2006-Apr. 2007 | 12.9 | n.d. | n.d. | Cao et al. (2009) |
|  | Suburban |  | 14.1 | n.d. | n.d. |  |
| Hunan, south China | Rural | Sep. 2010-Aug. 2008 | 2.6-6.7 | 13.5-20.0 | 2.7-3.2 | Shen et al. (2013) |
| 5 sites in south China | Rural | Jan. 2001-Dec. 2003 | n.d. | 1.9-18.5 | n.d. | Aas et al. (2010) |
| Jiangsu, south China | Rural | Oct. 2007-Sep. 2008 | 5.5 | 42.2 |  | Yang et al. (2010) |
| 31 Capital cities, China | Urban | Mar. 2013-Feb. 2014 | n.d. | 2.9-44.6 | n.d. | Wang et al. (2014b) |
| Clinton, Carolina, USA | Urban | Jan. 2000-Dec. 2000 | 5.5 | n.d. | n.d. | Walker et al. (2004) |
| Morehead, Carolina, USA | Urban | Jan. 2000-Dec. 2000 | 0.6 | n.d. | n.d. |  |
| Kinston, Carolina, UAS | Urban | May 2000-Dec. 2000 | 2.3 | n.d. | n.d. |  |
| Thessaloniki, Greece | Urban | Apr. 2002-Mar. 2003 | 2.3 | 31.0 | 0.8 | Anatolaki and Tsitouridou (2007) |
| Northern Adriatic | Urban | 1998-2005 | 12.5-20.7 | n.d. | n.d. | Alebic-Juretic (2008) |
| area, Croatia | Suburban |  | 6.1-28 | n.d. | n.d. |  |
| Agra, India | Suburban | Jul.-Sep. 1997 | 10.2 ± 6.4 | n.d. | n.d. | Singh et al. (2001) |
| Area, East Asia | Rural | Jan. 2007-Dec. 2007 | 1.0-4.5 | n.d. | n.d. | EANET (2007) |
| RondÔnia, Brazil | Rural | Sep.-Nov. 2002 | 0.5-1.8 | 1.1-4.6 | 0.1-0.3 | Trebs et al. (2006) |
| Tsukuba, Japan | Rural |  | 2.8 | n.d. | 1.8 | Hayashi et al. (2007) |
| Ijira, Japan | Rural | Apr. 2003-Mar. 2008 | 0.9 | n.d. | 0.9 | Endo et al. (2011) |
|  | Urban |  | 0.7 | n.d. | 0.8 |  |

^a^ no data

**References**

Aas W, Shao M, Jin L, Larssen T, Zhao DW, Xiang RJ et al (2007) Air concentrations and wet deposition of major inorganic ions at five non-urban sites in China, 2001–2003. Atmos Environ 41:1706–1716.

Alebic-Juretic A (2008) Airborne ammonia and ammonium within the Northern Adriatic area, Croatia. Environ Pollut 154:439–447.

Anatolaki Ch, Tsitouridou R (2007) Atmospheric deposition of nitrogen, sulfur and chloride in Thessaloniki, Greece. Atmos Res 91:413–428.

Cao JJ, Zhang T, Chow JC, Watson JG, Wu F, Li H (2009) Characterization of atmospheric ammonia over Xi’an, China. Aerosol and Air Qual Res 9:277–289.

EANET (2007) The Acid Deposition Monitoring Network in East Asia, Acid Deposition Monitoring Network in East Asia Date report 2006. Network Center for EANET. <http://www.eanet.cc/product.html>.

Endo T, Yagoh H, Sato K, Matsuda K, Hayashi K, Noguchi I et al (2011) Regional characteristics of dry deposition of sulfur and nitrogen compounds at EANET sites in Japan from 2003 to 2008. Atmos Environ 45:1259–1267.

Hayashi K, Komada M, Miyata A (2007) Atmospheric deposition of reactive nitrogen on turf grassland in central Japan: comparison of the contribution of wet and dry deposition. Water Air and Soil Poll 7:119–129.

Luo XS, Liu P, Tang AH, Liu JY, Zong XY, Zhang Q et al (2013) An evaluation of atmospheric N_r_ pollution and deposition in North China after the Beijing Olympics. Atmos Environ 74:209–216.

Meng ZY, Lin WL, Jiang XM, Yan P, Wang Y, Zhang YM et al (2011) Characteristics of atmospheric ammonia over Beijing, China. Atmos Chem Phys 11:6139–6151.

Shen JL, Tang AH, Liu XJ, Fangmeier A, Goulding KTW, Zhang FS (2009) High concentrations and dry deposition of reactive nitrogen species at two sites in the North China Plain. Environ Pollut 157:3106–3113

Shen JL, Li Y, Liu XJ, Luo XS, Tang AH, Zhang YZ et al (2013) Atmospheric dry and wet nitrogen deposition on three contrasting land use types of an agricultural catchment in subtropical central China. Atmos Environ 67:415–424.

Singh SP, Satsangi GS, Khare P, Lakhani A, Maharaj KK, Srivastava SS (2001) Multiphase Measurement of Atmospheric Ammonia. Chemosphere Global Change Science 3:107-116.

Treb I, Lara LL, Zeri LMM, Gatti LV, Artaxo P, Dlugi R, et al (2006) Dry and wet deposition of inorganic nitrogen compounds to a tropical pasture site (Rondônia, Brazil). Atmos Chem Phys 6:447–469.

Walker JT, Whitall D, Robarge WP, Paerl H (2004) Ambient ammonia and ammonium aerosol across a region of variable ammonia emission density. Atmos Environ 38:1235–1246.

Wang YG, Ying Q, Hu JL, Zhang HL (2014b) Spatial and temporal variations of six criteria air pollutants in 31 provincial capital cities in China during 2013–2014. Environ Int 73:413-422.

Yang R, Hayashi K, Zhu B, Li F, Yan X (2010) Atmospheric NH_3_ and NO_2_ concentration and nitrogen deposition in an agricultural catchment of Eastern China. Sci Total Environ 408:4624–4632.

**Table S4**. Summary statistics of daily average concentrations (μg m^-3^) of PM_2.5_ in each season from Mar. 2012 to Nov. 2014 at the four sites.

| Year |  | CAU |  |  |  |  | SZ |  |  |  |  | QZ |  |  |  |  | YC |  |  |  |
| --- | --- | --- | --- | --- | --- | --- | --- | --- | --- | --- | --- | --- | --- | --- | --- | --- | --- | --- | --- | --- |
|  |  | SP^a^ | SU | AU | WI |  | SP | SU | AU | WI |  | SP | SU | AU | WI |  | SP | SU | AU | WI |
| 2012/2013 | Mean | 168.2 | 110.8 | 154.0 | 203.8 |  | 323.7 | 81.4 | 107.9 | 121.4 |  | 155.3 | 102.0 | 157.3 | 68.1 |  | n.d. | n.d. | n.d. | n.d. |
|  | Med | 173.3 | 110.1 | 143.7 | 192.5 |  | 289.6 | 73.3 | 72.3 | 78.8 |  | 127.5 | 87.2 | 144.2 | 54.6 |  | n.d. | n.d. | n.d. | n.d. |
|  | Min | 24.9 | 11.8 | 20.6 | 51.9 |  | 81.9 | 31.1 | 19.8 | 29.9 |  | 69.6 | 48.2 | 45.9 | 33.4 |  | n.d. | n.d. | n.d. | n.d. |
|  | Max | 344.7 | 289.0 | 317.4 | 508.3 |  | 573.9 | 201.7 | 251.5 | 325.1 |  | 353.3 | 268.3 | 346.8 | 200.4 |  | n.d. | n.d. | n.d. | n.d. |
|  | Stdev | 79.6 | 65.4 | 81.5 | 102.1 |  | 170.8 | 37.9 | 67.9 | 86.7 |  | 78.2 | 47.0 | 76.6 | 43.7 |  | n.d. | n.d. | n.d. | n.d. |
|  | N | 25 | 28 | 30 | 32 |  | 7 | 23 | 26 | 31 |  | 12 | 30 | 30 | 14 |  | n.d. | n.d. | n.d. | n.d. |
| 2013/2014 | Mean | 198.3 | 89.7 | 152.0 | 206.7 |  | 105.5 | 84.4 | 176.5 | 218.6 |  | 174.5 | n.d. | 237.3 | 230.6 |  | 130.9 | 112.2 | 164.2 | 201.7 |
|  | Med | 186.2 | 76.6 | 133.3 | 182.9 |  | 86.3 | 74.0 | 128.5 | 186.0 |  | 147.9 | n.d. | 197.6 | 210.8 |  | 146.6 | 106.7 | 173.9 | 193.6 |
|  | Min | 73.1 | 38.7 | 29.4 | 53.2 |  | 25.9 | 29.7 | 48.0 | 42.3 |  | 43.9 | n.d. | 81.7 | 85.8 |  | 77.8 | 40.5 | 32.0 | 84.2 |
|  | Max | 477.5 | 273.1 | 336.0 | 456.2 |  | 274.3 | 189.5 | 692.9 | 503.2 |  | 436.9 | n.d. | 754.5 | 532.1 |  | 178.5 | 204.7 | 303.0 | 455.0 |
|  | Stdev | 91.2 | 48.6 | 77.3 | 94.1 |  | 57.5 | 40.8 | 124.5 | 116.4 |  | 85.4 | n.d. | 126.8 | 93.7 |  | 43.6 | 36.9 | 65.9 | 80.2 |
|  | N | 39 | 31 | 41 | 53 |  | 35 | 28 | 29 | 30 |  | 28 | n.d. | 35 | 34 |  | 5 | 16 | 27 | 33 |
| 2014/2015 | Mean | 221.8 | 78.7 | 141.4 | n.d. |  | 187.8 | 100.2 | 177.7 | n.d. |  | 149.5 | 72.4 | 118.2 | n.d. |  | 113.4 | 106.6 | 123.1 | n.d. |
|  | Med | 201.3 | 70.8 | 109.2 | n.d. |  | 166.2 | 88.4 | 118.9 | n.d. |  | 98.8 | 71.0 | 101.3 | n.d. |  | 117.8 | 97.7 | 113.7 | n.d. |
|  | Min | 84.2 | 25.0 | 18.7 | n.d. |  | 78.1 | 30.7 | 39.1 | n.d. |  | 48.0 | 51.5 | 23.9 | n.d. |  | 68.3 | 67.7 | 27.9 | n.d. |
|  | Max | 499.0 | 151.6 | 621.0 | n.d. |  | 387.6 | 262.6 | 651.0 | n.d. |  | 472.8 | 106.3 | 438.1 | n.d. |  | 182.3 | 176.9 | 360.3 | n.d. |
|  | Stdev | 102.4 | 38.3 | 108.3 | n.d. |  | 82.9 | 46.4 | 152.5 | n.d. |  | 111.1 | 12.4 | 80.6 | n.d. |  | 32.0 | 27.3 | 71.3 | n.d. |
|  | N | 26 | 30 | 49 | n.d. |  | 28 | 31 | 31 | n.d. |  | 32 | 30 | 25 | n.d. |  | 18 | 30 | 26 | n.d. |

^a^ SP = spring; SU = summer; AU = autumn; WI = winter.

n.d. = no data.

**Table S5**. Pearson correlation coefficients between molar concentrations of NH_4_^+^, NO_3_^-^, SO_4_^2-^ and Cl^-^ in PM_2.5_ at the four sites.

| Site | No | NO_3_^-^ vs NH_4_^+^ |  | SO_4_^2-^ vs NH_4_^+^ |  | Cl^-^ vs NH_4_^+^ |  | (NO_3_^-^+SO_4_^2-^) vs NH_4_^+^ |  | (NO_3_^-^+SO_4_^2-^+Cl^-^) vs NH_4_^+^ |
| --- | --- | --- | --- | --- | --- | --- | --- | --- | --- | --- |
|  |  | PM_2.5_ |  | PM_2.5_ |  | PM_2.5_ |  | PM_2.5_ |  | PM_2.5_ |
| CAU | 384 | 0.904^**^ |  | 0.892^**^ |  | 0.476^**^ |  | 0.952^**^ |  | 0.938^**^ |
| SZ | 299 | 0.840^**^ |  | 0.848^**^ |  | 0.523^**^ |  | 0.901^**^ |  | 0.905^**^ |
| QZ | 270 | 0.843^**^ |  | 0.848^**^ |  | 0.449^**^ |  | 0.926^**^ |  | 0.919^**^ |
| YC | 155 | 0.854^**^ |  | 0.855^**^ |  | 0.467^**^ |  | 0.934^**^ |  | 0.926^**^ |

^**^ significant at *p*<0.01.

**Table S6**. Seasonal SO_2_ concentrations (μg m^-3^)^a^ for sampling periods at CAU, SZ and QZ

|  | Spring | Summer | Autumn | Winter |
| --- | --- | --- | --- | --- |
| CAU | 19.4 | 8.4 | 25.0 | 67.1 |
| SZ | 13.4 | 6.3 | 10.5 | 36.5 |
| QZ | 26.9 | 17.8 | 31.1 | 56.5 |

^a^ The monthly average SO_2_ data were monitored using the DELTA system from Jan. 2011 to Jul. 2012 at CAU, Jan. 2011 to Aug. 2012 at SZ, and Jan.11 to Feb. 2012 at QZ.
